# Supplementary material for: Plasma Protein Biomarkers Distinguish Multisystem Inflammatory Syndrome in Children From Other Pediatric Infectious and Inflammatory Diseases
Source: Pediatr Infect Dis J. 2024 Feb 7;43(5):444–53. doi: 10.1097/INF.0000000000004267 (PMC11003410; doi:10.1097/INF.0000000000004267)

**Supplemental Digital Content 1.** Breakdown of causative agents identified in the Definite Bacterial and Definite Viral groups.


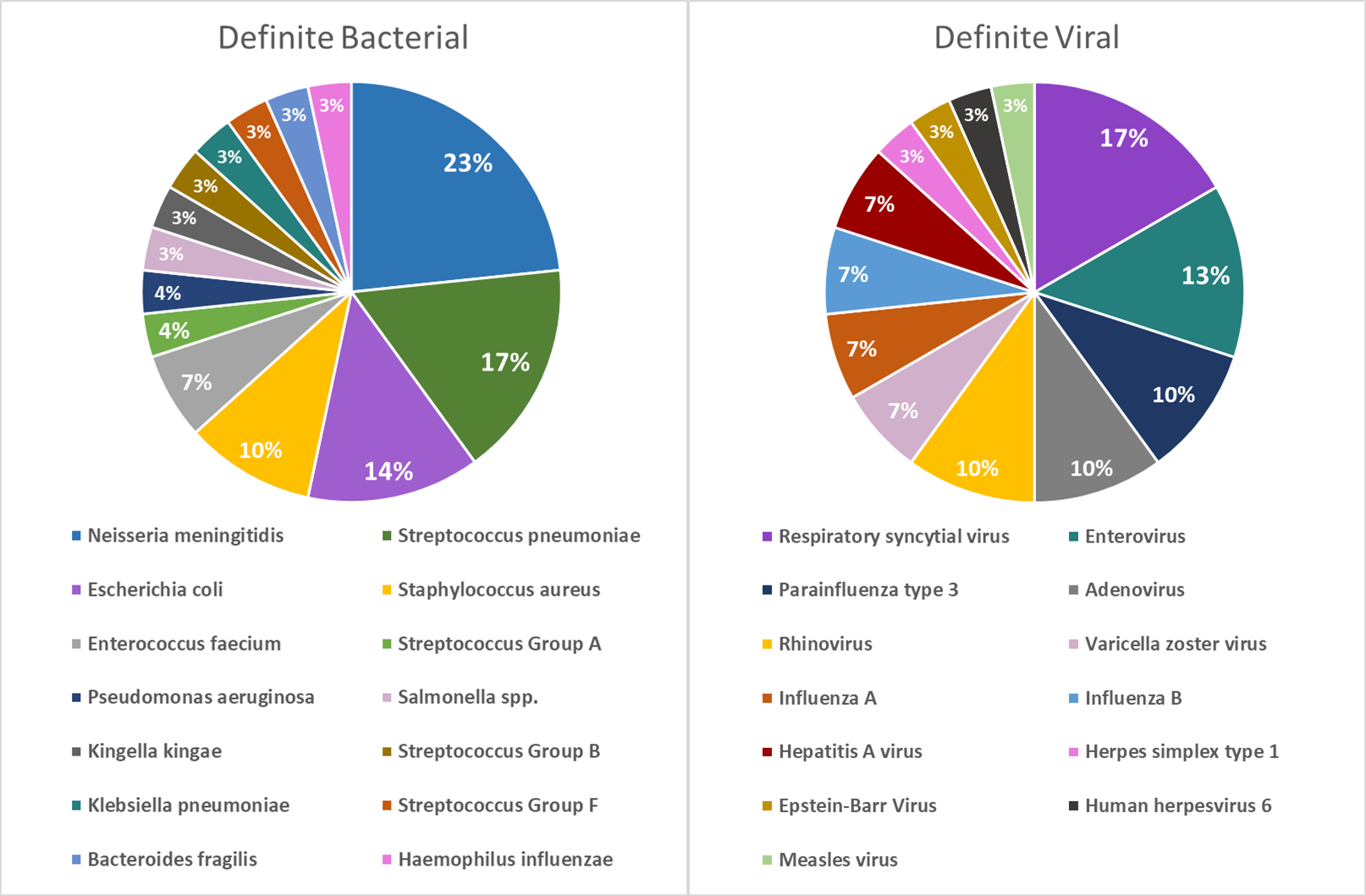

Supplement: Supplementary file 1 [file inf-43-0444-s001.docx]
