# Supplementary material for: Plasma Protein Biomarkers Distinguish Multisystem Inflammatory Syndrome in Children From Other Pediatric Infectious and Inflammatory Diseases
Source: Pediatr Infect Dis J. 2024 Feb 7;43(5):444–53. doi: 10.1097/INF.0000000000004267 (PMC11003410; doi:10.1097/INF.0000000000004267)
Supplement: Supplementary file 2 [file inf-43-0444-s002.docx]

**Supplemental Digital Content 2.** Proteins selected for analysis, their function and reason for inclusion. LFC= Log-fold change. P= adjusted *p*-value.

| **Protein** | **UniProt ID** | **Function** | **Justification for inclusion** | **Relevant publication(s)** |
| --- | --- | --- | --- | --- |
| ADAMTS2 | O95450 | Metalloprotease cleaving procollagens | RNA-Seq analysis:  LFC 3.42, p= 3.03 E-09 |  |
| ARG1 | O95450 | Hydrolase in urea cycle | RNA-Seq analysis:  LFC 2.31, p= 9.47 E-12 |  |
| CCL20 | P78556 | Inflammation and homing chemokine | Literature search | (6) |
| CD163 | Q86VB7 | Haemoglobin scavenger receptor | RNA-Seq analysis:  LFC 2.66, p=4.34 E-18 |  |
| CORIN | Q9Y5Q5 | Serine protease responsible atrial natriuretic peptide (ANP) production | RNA-Seq analysis:  LFC 2.75, p= 3.79 E-20 |  |
| CXCL9 | Q07325 | Chemokine involved in inflammatory response | Literature search | (28, 17) |
| PCSK9 | Q8NBP7 | Low-density lipoprotein (LDL) receptor regulation | RNA-Seq analysis:  LFC 4.94, p= 3.10 E-08 |  |
