# Supplementary material for: Plasma Protein Biomarkers Distinguish Multisystem Inflammatory Syndrome in Children From Other Pediatric Infectious and Inflammatory Diseases
Source: Pediatr Infect Dis J. 2024 Feb 7;43(5):444–53. doi: 10.1097/INF.0000000000004267 (PMC11003410; doi:10.1097/INF.0000000000004267)

**Supplemental Digital Content 3.** Principal component analysis (PCA) biplots generated from the protein abundance values of the 7 proteins measured in this study. Principal component (PC) 1 and 2 are shown. Points are coloured by disease group (A), sex (B), and age in years (C).


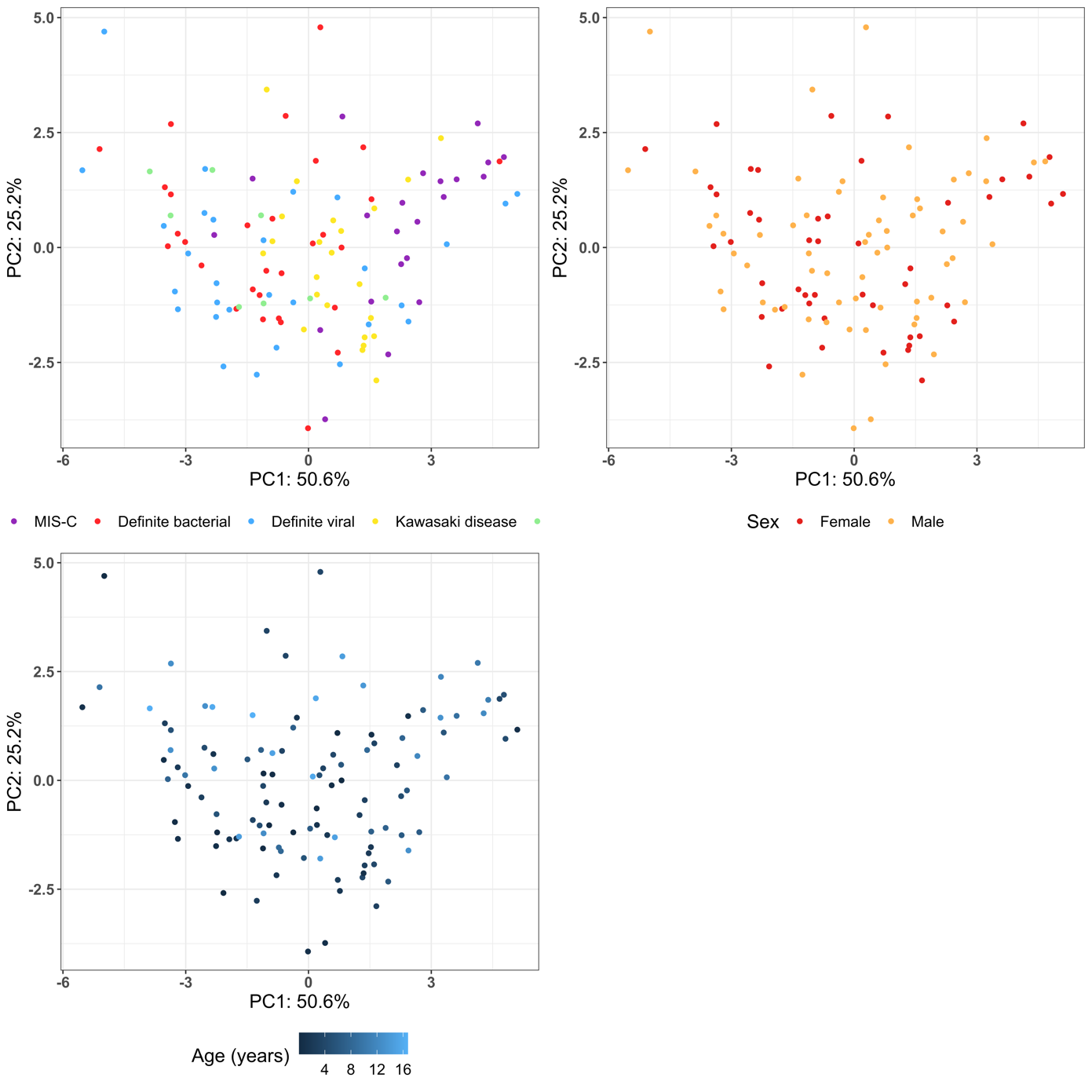

Supplement: Supplementary file 3 [file inf-43-0444-s003.docx]
