# Supplementary material for: Plasma Protein Biomarkers Distinguish Multisystem Inflammatory Syndrome in Children From Other Pediatric Infectious and Inflammatory Diseases
Source: Pediatr Infect Dis J. 2024 Feb 7;43(5):444–53. doi: 10.1097/INF.0000000000004267 (PMC11003410; doi:10.1097/INF.0000000000004267)
Supplement: Supplementary file 4 [file inf-43-0444-s004.docx]

**Supplemental Digital Content 4.** Significance levels of each of the proteins measured. *P*-values obtained from the Wilcoxon test for the pairwise comparisons and Kruskal-Wallis test for MIS-C *vs.* all groups, with all *p*-values corrected using the Bonferroni adjustment. MIS-C = multisystem inflammatory syndrome in children; KD = Kawasaki disease; DB = definite bacterial; DV = definite viral; HC = healthy control. Significant *p*-values are shown in underlined and bold font.

| Protein | MIS-C *vs*. KD, DB, DV, HC | MIS-C *vs*. KD | MIS-C *vs*. DB | MIS-C *vs*. DV |
| --- | --- | --- | --- | --- |
| PCSK9 | **2.1x10^-3^** | **8.7x10^-3^** | 0.056 | **1.4x10^-2^** |
| CD163 | **8.010^-6^** | **0.014** | **4.2x10^-4^** | **2.7x10^-5^** |
| CXCL9 | **1.3x10^-5^** | **0.029** | **6.2x10^-5^** | **8.4x10^-4^** |
| CORIN | **4.8x10^-2^** | 0.910 | 1.0 | **0.022** |
| ARG1 | 0.480 | 1.0 | 0.132 | 1.0 |
| CCL20 | 1.0 | 1.0 | 1.0 | 1.0 |
| ADAMTS2 | 0.676 | 1.0 | 1.0 | 1.0 |
