# Supplementary material for: Plasma Protein Biomarkers Distinguish Multisystem Inflammatory Syndrome in Children From Other Pediatric Infectious and Inflammatory Diseases
Source: Pediatr Infect Dis J. 2024 Feb 7;43(5):444–53. doi: 10.1097/INF.0000000000004267 (PMC11003410; doi:10.1097/INF.0000000000004267)
Supplement: Supplementary file 5 [file inf-43-0444-s005.docx]

**Supplemental Digital Content 5.** The performance of all possible combinations of PCSK9, CD163 and CXCL9 in distinguishing MIS-C from DB, DV and KD. *: “All” includes DB, DV and KD. AUCs and 95% confidence intervals are shown in the table. **: for this comparison, MIS-C vs. all represents MIS-C vs. DB and KD as CRP was used in the classification of DV patients. ***: perfect classification is expected as CRP was used in the classification of DV samples.

| **Combination** | **MIS-C *vs.* all*** | **MIS-C *vs.* DB** | **MIS-C *vs.* DV** | **MIS-C *vs.* KD** |
| --- | --- | --- | --- | --- |
| PCSK9+CD163+CXCL9 | 86.9% (76.8%-95.1%) | 87.5% (77.2%-97.9% | 86.7% (76.3%-97.1%) | 83.2% (69.9%-96.5) |
| CD163+PCSK9 | 85.0% (75.7%-94.3%) | 81.8% (69.1%-94.5%) | 88.8% (79.7%-97.9%) | 84.0% (72.1%-95.9%) |
| CD163+CXCL9 | 84.3% (75.0%-93.7%) | 86.5% (75.9%-97.2%) | 86.4% (75.9%-96.9%) | 79.3% (64.2%-94.3%) |
| PCSK9+CXCL9 | 82.6% (72.0%-93.2%) | 86.5% (76.2%-96.9%) | 82.5% (70.4%-94.5%) | 78.1% (62.9%-93.2%) |
| PCSK9 | 74.2% (61.3%-87.2%) | 69.7% (54.4%-85.0%) | 75.3% (61.1%-89.5%) | 78.3% (64.4%-92.1%) |
| CD163 | 82.8% (74.0%-91.5%) | 81.1% (68.5%-93.8%) | 88.8% (79.8%-97.8%) | 77.3% (63.5%-91.0%) |
| CXCL9 | 81.4% (71.0%-91.9%) | 85.0% (74.1%-96.0%) | 82.8% (71.0%-94.6%) | 75.5% (59.5%-91.5%) |
| 3-proteins + CRP** | 81.1% (71.3%-90.9%) | 71.5% (56.9%-86.2%) | 100% *** | 92.3% (84.8%-99.8%) |
