# Supplementary material for: Plasma Protein Biomarkers Distinguish Multisystem Inflammatory Syndrome in Children From Other Pediatric Infectious and Inflammatory Diseases
Source: Pediatr Infect Dis J. 2024 Feb 7;43(5):444–53. doi: 10.1097/INF.0000000000004267 (PMC11003410; doi:10.1097/INF.0000000000004267)
Supplement: Supplementary file 6 [file inf-43-0444-s006.docx]

**Supplemental Digital Content 6:** A breakdown of clinical groups and recruitment sites for patients admitted to PICU, oxygen requirement, invasive and non-invasive ventilation and inotrope requirement. (**SMH**: St. Mary’s Hospital, London; **UOXF**: John Radcliffe Hospital, Oxford; **SCH**: Sheffield Children’s Hospital, Sheffield; **LCH**: Leed’s Children’s Hospital, Leeds; **LIV**: Alder Hey Children’s Hospital Trust, Liverpool; **UNEW**: Newcastle upon Tyne Hospitals Foundation Trust, Newcastle upon Tyne; **NKUA**: Panagiotis and Aglaia Kyriakou Hospital, Athens, Greece; **RUMC**: Radboud University Medical Center, Nijmegen, The Netherlands)

|  | **MIS-C** | **DB** | **DV** | **KD** |
| --- | --- | --- | --- | --- |
| **PICU admission** | | | | |
| **Total number PICU** | 12 | 6 | 16 | 1 |
| **PICU split by site** | SMH n=7; UOXF n=1; SCH n=3; LCH n=1 | SMH n=1; LIV n=2; UNEW n=2; NKUA n=1 | SMH n=12; UNEW n=1; RUMC n=1; NKUA n=2 | SMH n=1 |
| **Oxygen requirement** | | | | |
| **Total number oxygen** | 3 | 9 | 14 | 0 |
| **Oxygen split by site** | SMH n=1; UOXF n=1; SCH n=1 | SMH n=4; LIV n=2; UNEW n=2; RUMC n=1 | SMH n=12, UNEW n=1, NKUA n=1 |  |
| **Non-invasive ventilation requirement** | | | | |
| **Total number non-invasive ventilation** | 2 | 1 | 9 | 0 |
| **Non-invasive ventilation split by site** | SMH n=1; SCH n=1 | RUMC n=1 | SMH n=9 |  |
| **Invasive ventilation requirement** | | | | |
| **Total number invasive ventilation** | 1 | 7 | 15 | 0 |
| **Invasive ventilation split by site** | UOXF n=1 | SMH n=4; LIV n=2; UNEW n=1 | SMH n=12; LIV n=1; UNEW n=1; RUMC n=1 |  |
| **Inotrope requirement** | | | | |
| **Total number inotropes** | 7 | 4 | 3 | 0 |
| **Inotropes split by site** | SMH n=5; UOXF n=1; SCH n=1 | LIV n=2; SMH n=1; UNEW n=1 | SMH n=3 | 0 |
